# Shedding Light on Treatment Options for Coronary Vasomotor Disorders: A Systematic Review

**DOI:** 10.1007/s10557-022-07351-x

**Published:** 2022-06-09

**Authors:** Federico Marchini, Graziella Pompei, Emanuele D’Aniello, Andrea Marrone, Serena Caglioni, Simone Biscaglia, Gianluca Campo, Matteo Tebaldi

**Affiliations:** Cardiology Unit, Azienda Ospedaliero Universitaria Di Ferrara, Via Aldo Moro 8, 44124 Cona, FE Italy

**Keywords:** Microvascular angina, Vasospastic angina, Non obstructive coronary artery disease, Vasomotor disorders

## Abstract

**Purpose:**

Coronary vasomotor dysfunction embraces two specific clinical entities: coronary (micro)vascular spasm and microvascular dysfunction. The clinical manifestations of these entities are respectively called vasospastic angina (VSA) and microvascular angina (MVA). Over the years, these diseases have become more and more prominent and several studies aimed to investigate the best diagnostic and therapeutic strategies. Patients with coronary vasomotor disorders are often undertreated due to the absence of evidence-based guidelines. The purpose of this overview is to illustrate the various therapeutic options available for the optimized management of these patients.

**Methods:**

A Medline search of full-text articles published in English from 1980 to April 2022 was performed. The main analyzed aspects of vasomotor disorders were treatment options. We also performed research on “Clinicaltrial.gov” for ongoing trials.

**Conclusion:**

Coronary (micro)vascular spasm and microvascular dysfunction are clinical entities characterized by high prevalence and clinical representation. Several therapeutic strategies, both innovative and established, are available to optimize treatment and improve the quality of life of these patients.

## Introduction

The term coronary vasomotor dysfunction encompasses two specific clinical entities: coronary (micro)vascular spasm and microvascular dysfunction. The clinical manifestations of these entities are called, respectively, vasospastic angina (VSA) and microvascular angina (MVA). In the last years, these endotypes have had more and more prominence thanks to an increasing number of studies that have investigated not only the physiopathological and diagnostic entity, but also the therapeutic strategies.

Many patients with chest pain enrolled for coronary angiography do not show significant obstructive coronary artery disease (CAD). The rate of these patients is variable according to the studies. Patel MR et al. demonstrated that nearly 60% of symptomatic patients undergoing coronary angiography have non-obstructive CAD [1]. A retrospective registry from Eastern Denmark including 11,223 patients undergoing coronary angiography for angina between 1998 and 2009 showed that 65% of women and 33% of men had non-obstructive CAD, with an increasing rate over 10 years in both sexes [2, 3]. Moreover, 62% of women enrolled in the National Heart, Lung and Blood institute-sponsored Women’s Ischemia Syndrome Evaluation (WISE), who were referred for coronary angiography, did not show obstructive CAD [4].

**Fig. 1:**
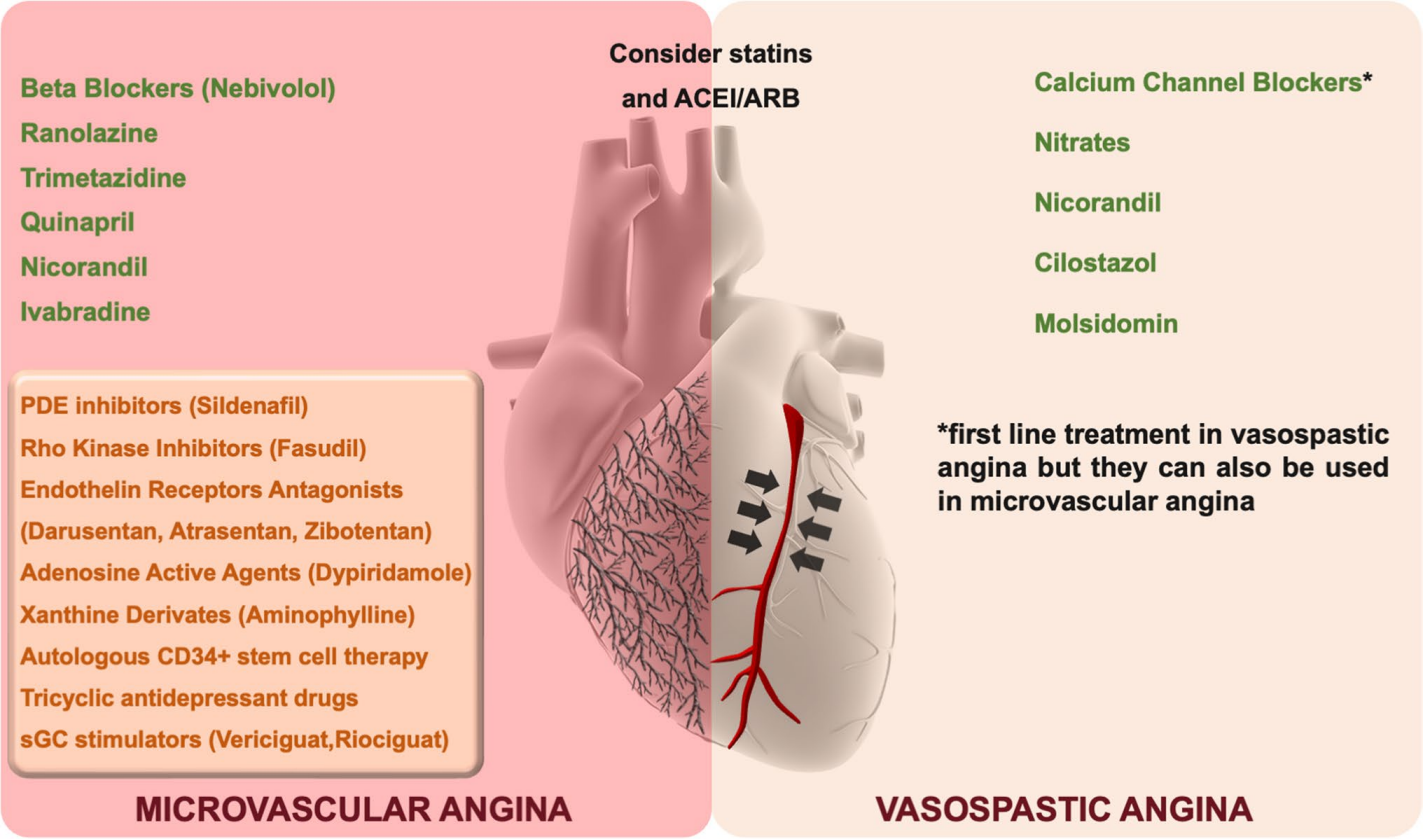
Therapeutic management of vasomotor disorders. In green, currently available medications. In orange, the drugs under study

Just from the therapeutic point of view, there are numerous pharmacological approaches often not considered for the management of these diseases (Fig. 1). The purpose of this overview is to illustrate the various therapeutic options available for the optimized management of these patients.

## Brief Methodological Considerations

A Medline search of full-text articles published in English from 1980 to April 2022 was performed. Overall, 20,118 records were identified. The search terms were the following: ((microvascular dysfunction) OR (microvascular disease) OR (coronary spasm) AND (treatment). Only papers published in English and peer-reviewed journals were selected. The main analyzed aspects of vasomotor disorders were treatment options. After evaluation of title and abstract, a total of 112 studies were analyzed as full text. The quality of selected papers was tested using MINORS criteria. Unblinded reviewers performed the analysis of full texts for quality assessment. Discrepancies between reviewers have been solved by consensus. The maximum score obtained was 14 and the minimum 8. We included in the present review only studies obtaining a score 10. A total of 66 papers were then considered for this overview. We also performed research on “Clinicaltrial.gov” for ongoing trials.

## Pathophysiology

The heart has limited anaerobic capacity and, in resting condition, its oxygen extraction is almost 75%. Therefore, an increased oxygen demand can be met only by adjustment of coronary blood flow [5].

This mechanism may be compromised either by vascular spasm or impaired microvascular dilatation. Both phenomena could occur at epicardial level or microvascular level, compromising the ability to regulate the coronary blood flow in response to the increased myocardial oxygen demand.

Vascular spasm is relevant both in epicardial vessels and microcirculation: in fact, a sudden excessive coronary vasoconstriction produces a transient reduction of blood flow, resulting in myocardial ischemia. The clinical manifestation of myocardial ischemia caused by dynamic epicardial obstruction is called epicardial vasospastic angina. This term covers several forms of vasomotor disorders caused by epicardial vessels spasm and characterized by chest pain and ECG ischemic changes. Vasospastic angina, however, may also be the result of a microvascular spasm.

Impaired vasodilatation, on the other hand, most significantly affects coronary microcirculation because the epicardial coronary arteries are mainly conductance vessels and give little contribution to coronary resistance [6]. At this level, structural remodeling of the microvasculature (leading to fixed reduced microcirculatory conductance) or vasomotor disorders affecting the coronary arterioles (causing dynamic arteriolar obstruction) may result in myocardial ischemia [3].

Furthermore, the coronary vasomotor tone may be altered either by endothelium-dependent or endothelium-independent factors. In normal conditions, the endothelium releases vasodilator molecules to preserve an appropriate dilatation in response to physiological stimuli. Compromising the balance between both endothelium relaxing and constriction factors leads to the endothelium-dependent dysfunction. On the other hand, endothelium-independent function directly involves the myocyte tone. The myocyte tone is regulated by several stimuli (e.g., autonomic nervous system, adenosine, intracellular G-proteins, and enzymes) and the dysfunction of these systems leads to vascular smooth cell hyperreactivity [7].

The clinical manifestation of myocardial ischemia caused by microvascular dysfunction or microvascular spasm is called microvascular angina and is the result of the structural remodeling of microvasculature and/or vasomotor disorders affecting the coronary arterioles.

Both microvascular and vasospastic angina can occur in the same patient, leading to a worse prognosis [8].

## Diagnosis of Coronary Vasomotor Disorders

A detailed description of the diagnostic flow and diagnostic tools is outside the aim of the present systematic review. A more comprehensive discussion can be found in the following references [9, 10].

Coronary vascular dysfunction can be assessed with an invasive coronary reactivity test, evaluating coronary flow reserve (CFR), microvascular resistance, and the presence of macrovascular and microvascular coronary artery spasms. First, it is mandatory to rule out an obstructive CAD; afterwards, both macrovascular and microvascular functions need to be evaluated.

The diagnosis of vasospasm can be made with a provocative test (typically using acetylcholine), which is considered positive in case of reproduction of recognizable symptoms (such as chest pain or dyspnea), and ischemic ECG changes and ≥ 90% constriction of epicardial vessels at coronary angiography.

For the diagnosis of microvascular spasm, coronary flow needs to be reduced or increased in the absence of epicardial coronary artery spasm. The diameter of the coronary diameter is maintained in association with transient reduction of coronary flow (TIMI flow grade less than 3), while the patient generally experiences symptoms in association with ischemic changes on electrocardiography [10].

CFR and microvascular resistance, instead, are measured using adenosine and a thermodilution-based or Doppler-based wire. Coronary microvascular dysfunction is defined as impaired CFR (cutoff < 2 or 2.5 depending on the methodology used), anomalous microvascular resistance (IMR ≥ 25 or hMR > 2.4 mmHg/cm), or slow flow phenomenon [11].

## Covadis Criteria

Diagnostic criteria for both epicardial VSA and MVA have been proposed by Coronary Vasomotion Disorders International Study Group (COVADIS).

## VSA Diagnostic Criteria

1. Nitrate-responsive angina—during spontaneous episode, with at least one of the following:
  - rest angina—especially between night and early morning;
  - marked diurnal variation in exercise tolerance—reduced in morning;
  - hyperventilation can precipitate an episode;
  - calcium channel blockers suppress episodes

(2) Transient ischemic ECG changes—during spontaneous episodes, including any of the following in at least two contiguous leads:
  - ST segment elevation ≥0.1 mV
  - ST segment depression ≥0.1 mV
  - New negative U waves

(3) Coronary artery spasm—defined as transient total or subtotal coronary artery occlusion (at least 90% constriction) with angina and ischemic ECG changes either spontaneously or in response to a provocative stimulus (typically acetylcholine, ergot, or hyperventilation).

The diagnosis of vasospastic angina may be considered *definitive* if nitrate-responsive angina is evident during spontaneous episodes and either the transient ischemic ECG changes during the spontaneous episodes or coronary artery spasm criteria are fulfilled.

The diagnosis of vasospastic angina, instead, is *suspected* if nitrate-responsive angina is evident during spontaneous episodes, but transient ischemic ECG changes are equivocal or unavailable and coronary artery spasm criteria are equivocal [12].

## MVA Diagnostic Criteria

1. Symptoms of myocardial ischemia (effort and/or rest angina; angina equivalents);
2. Absence of obstructive CAD (< 50% diameter reduction or FFR > 0.80) by coronary computed tomography or invasive coronary angiography.
3. Objective evidence of myocardial ischemia (ischemic ECG changes during an episode of chest pain; stress-induced chest pain and/or ischemic ECG changes in the presence or absence of transient/reversible abnormal myocardial perfusion and/or wall motion abnormality).
4. Evidence of impaired coronary microvascular function, which includes:
  - Impaired coronary flow reserve (cut-off values depending on methodology used between ≤ 2.0 and ≤ 2.5)
  - Coronary microvascular spasm, defined as the reproduction of symptoms, ischemic ECG shifts but no epicardial spasm during acetylcholine testing.
  - Abnormal coronary microvascular resistance indices (e.g., IMR > 25)
  - Coronary slow flow phenomenon, defined as TIMI frame count > 25 [13].

The diagnosis of MVA is considered *definitive* if all four criteria are present.

The diagnosis MVA is *suspected* if symptoms of ischemia are present (criteria 1) with no obstructive coronary artery disease (criteria 2) but only (a) objective evidence of myocardial ischemia (criteria 3), or (b) evidence of impaired coronary microvascular function (criteria 4) alone.

## Therapy

### Lifestyle and Cardiovascular Risk Factors Management

The management of vasomotor disorders is quite challenging since, as of today, there is no evidence-based treatment. First, patients should be encouraged to act on their lifestyle, change their nutrition habits, exercise, and focus on weight reduction, completely stop smoking and focus on stress management. Furthermore, it is crucial to manage the main cardiovascular risk factors, such as hypertension, dyslipidemia, and diabetes mellitus. Reducing high blood pressure has proven to be effective in preventing the progression of microvascular dysfunction, and the use of ACE-inhibitors (ACE-I) may be proposed to improve CFR in microvascular dysfunction [14]. In addition, the anti-inflammatory effect of statins may be useful to improve CFR and reduce vascular spasms [15, 16]. The ongoing WARRIOR study (ClinicalTrials.gov NCT03417388), will assess the benefits of medical therapy with high-intensity statin, ACE-I, and aspirin on long-term outcomes in a population of women with chest pain and non-obstructive coronary artery disease.

### *Epicardial Vessel Spasm–Vasospastic Angina* (Table 1).

**Table 1:** Currently available drugs and clinical indication

Disease	Drugs
Microvascular angina	Beta-blockers (nebivolol 2.5–10 mg daily)
	Calcium channel blockers (amlodipine 10 mg daily)
	Ranolazine (375–750 mg twice daily)
	Trimetazidine (35 mg twice daily)
	Nicorandil (10–20 mg twice daily)
	Ivabradine (5–7.5 mg twice daily)
	Quinapril (10–40 mg daily)
Both microvascular angina and vasospastic angina	Consider ACE-inhibitors (ramipril 2.5–10 mg daily) and statins (rosuvastatin 10–20 mg)
Vasospasticangina	Calcium channel blockers (amlodipine 10 mg; verapamil 240 mg single dose; diltiazem 90 mg twice daily or 120–360 mg single or divided doses; benidipine 10–30 mg, nifedipine 30–60 mg)
	Nicorandil (10–20 mg twice daily)
	Long-acting nitrates (isosorbide mononitrate XL 30 mg)

#### Calcium Channel Blockers

Calcium channel blockers (CCBs) are the first-line treatment for vasospastic angina (amlodipine 10 mg or verapamil 240 mg single dose or diltiazem 90 mg twice daily or 120–360 mg single or divided doses). They are known to reduce spontaneous and inducible coronary spasms via vascular smooth muscle relaxation and to act on cardiac oxygen demand. In patients with severe VSA, high dosages of calcium channel blockers may be needed or even a combination of non-dihydropyridine with dihydropyridine calcium blockers [17, 18]. CCBs have the most important role in both endotypes of coronary vasospasm and in microvascular dysfunction; however, solid evidence is lacking. The recent “Efficacy of Diltiazem to improve coronary vasomotor dysfunction in angina and non-obstructive coronary arteries (EDT-CMD)” trial studied the effect of diltiazem in a population of 85 patients suffering from angina, non-obstructive CAD, and confirmation of coronary vascular dysfunction (defined as the presence of vasospasm after intracoronary acetylcholine provocation and/or microvascular dysfunction with CFR < 2 and/or IMR > 25). After 6 weeks, diltiazem failed to improve coronary function testing, microvascular dysfunction, symptoms (SAQ), or quality of life (RAND-36). However, more patients on diltiazem treatment progressed from epicardial spasm to microvascular or no spasms (47% vs. 6%, *p* = 0.006) [19].

Previous small trials demonstrated a favorable effect of diltiazem, in contrast to EDIT-CMD. However, those studies were performed on a different population, small sample sizes without differentiation of vasomotor dysfunction endotypes or including patients with CAD and many of them were not placebo-controlled [20, 21].

The different effect of first-generation CCBs (diltiazem and nifedipine) vs second-generation CCBs (amlodipine and benidipine) has been evaluated in 1586 patients enrolled in the Vasospastic Angina in Korea (VA-KOREA) registry. In this registry, VSA was defined as a total (100%) or subtotal (> 90% luminal diameter narrowing) occlusion of the index coronary artery accompanied with ischemic symptoms and/or ECG changes. There was no difference in the primary outcome (time to events of the composite of death from any cause, acute coronary syndrome, and symptomatic arrhythmias during 3-year follow-up). However, the incidence of acute coronary syndrome was lower in the 2nd-generation CCBs group with a person-month incidence rate of 1.66 vs. 0.35 (HR, 0.22; 95% CI, 0.05 to 0.89; *p* = 0.034). The use of benidipine showed a better control of angina symptom compared with diltiazem for 3 years (odds ratio, 0.17; 95% CI, 0.09 to 0.32; *p* < 0.0001 at 3rd year) [22]. Moreover, in a meta-analysis performed by Minatogugchi, benidipina showed a significant better prognostic effect on major acute cardiovascular events (MACE) than amlodipine, nifedipine, or diltiazem [23].

The effect of intracoronary (IC) CCBs has been studied also in the coronary slow-flow phenomenon. This phenomenon is characterized by delayed coronary opacification during diagnostic angiography in the absence of epicardial CAD and it may be associated with microvascular spasms [24]. In a study of 30 patients suffering from angina with angiography evidence of the slow-flow phenomenon, IC nicardipine produced markedly accelerated coronary filling, which was corroborated by TIMI frame count analysis (TIMI frame count 47 ± 17 before vs 15 ± 5 after nicardipine, *p* < 0.001) [25].

#### Nitrates

For more severe or refractory symptoms, guidelines suggest the addition of long-acting nitrates because they may have some coronary vasodilatory effect. However, the clinical benefit of this approach is controversial. In fact, the prognostic impact of vasodilated therapy has recently been investigated in patients with VSA from the multicenter, prospective VA-KOREA registry. Patients were divided into four groups: no vasodilators, non-nitrate vasodilators, conventional nitrates, and a combination of conventional nitrates and other vasodilators. The results showed no difference in primary endpoint (a composite of cardiac death, acute coronary syndrome, and new-onset arrhythmia at 2 years) between no vasodilators group and vasodilator groups. However, the acute coronary syndrome risk was significantly higher in the conventional nitrate (hazard ratio [HR], 2.49; 95% CI, 1.01–6.14; *P* = 0.047) and combination groups (HR, 3.34; 95% CI, 1.15–9.75, *P* = 0.027) compared with the no-vasodilator group, especially in low-risk patients [26]. Two previous studies of vasodilator therapy in VSA (defined as at least 90% diameter stenosis of the coronary arteries after intracoronary ergonovine injection in addition to ischemic symptoms and/or electrocardiographic changes) showed similar results: in the first one, retrospective data from 1429 patients with VSA indicated that chronic nitrate therapy in combination with nicorandil was associated with a two-fold increase in cardiac events (HR: 2.14; 95% CI: 1.02–4.47; P = 0.044) [27]. In the second observational study including data from 1154 patients with VSA and positive ergonovine provocative test, nitrate therapy increased the risk of a composite of cardiac death, myocardial infarction, revascularization, and rehospitalization due to recurrent angina [28].

#### Nicorandil

Another vasodilator drug is nicorandil, which is a combinatorial vasodilator agent acting via nitrate and potassium channel activation. Even if guidelines mention it as a possible effective therapy [3], prior studies showed a neutral effect on clinical outcomes [26, 27].

#### Molsidomin

Molsidomin is a long-acting vasodilator which has proved to shorten the duration of exercise-induced angina and reduce ST-segment depression measured by ECG [29]. Moreover, in a small study of 10 patients with angina and coronary spasm induced by alkalosis, Molsidomin was able to prevent the development of coronary artery spasm in 8 of 10 patients [30].

#### Cilostazol

Cilostazol is an inhibitor of phosphodiesterase 3. It has vasodilatation properties, and antiplatelet action and it is able to inhibit vascular smooth cell growth. Its efficacy and safety in VSA (defined as at least 90% diameter stenosis of the coronary arteries after intracoronary ergonovine injection in addition to ischemic symptoms and/or electrocardiographic changes) have been studied in the STELLA trial (Study to evaluaTe the Efficacy and safety of Pletaal ciLostazoL in subjects with vAsospastic angina). In this trial, cilostazol was an effective therapy for patients with uncontrolled VSA: in fact, the primary endpoint (relative reduction of chest pain frequency during the last week of the double-blind period compared to the 1-week period before randomization) was significantly greater in the cilostazol group compared with the placebo group (− 66.5 ± 88.6% vs − 17.6 ± 140.1%, respectively, *p* = 0.009). The secondary endpoints include a change in the frequency of chest pain (− 3.7 ± 0.5 vs − 1.9 ± 0.6, respectively, *p* = 0.029), a change in the chest pain severity scale (− 2.8 ± 0.4 vs − 1.1 ± 0.4, respectively, *p* = 0.003), and the proportion of chest pain-free patients (76.0% vs 33.3%, respectively, *p* = 0.003) also significantly favored cilostazol [31].

### *Microvascular angina* (Table 1, Table 2).

**Table 2:** All drugs studied and trials currently underway for the management of vasomotor disorders

Drugs	Indication	Ongoing trials
Calcium channel blockers (amlodipine, verapamil, diltiazem, nifedipine, benidipine, nicardipine)	First line treatment in vasospastic angina Can be used in microvascular angina	
Nitrates	Vasospastic angina	
Cilostazol	Vasospastic angina	
Molsidomin	Vasospastic angina	
Nicorandil	Vasospastic angina + Microvascular angina	
Beta Blockers (nebivolol)	Microvascular angina	**NIRVANA** (NCT016655089)
Ranolazine	Microvascular angina	
Quinapril	Microvascular angina	
Ivabradine	Microvascular angina	
PDE inhibitors (sildenafil)	Microvascular angina	
Vericiguat and riociguat	Microvascular angina	
Rho kinase inhibitors (fasudil)	Microvascular angina	
Endothelin receptors antagonist (darusentan, atrasentan, zibotentan)	Microvascular angina	**PRIZE**(NCT04097314)
Adenosine active agents (dypiridamole)	Microvascular angina	
Xanthine derivates (aminophylline)	Microvascular angina	
Trimetazidine	Microvascular angina	
Autologous CD34 + stem cell therapy	Microvascular angina	
Tricyclic antidepressant drugs	Microvascular angina	
ACE inhibitors and statins	Vasospastic angina + Microvascular angina	**WORRIOR** (NCT03417388)

#### Beta-blockers

Patients suffering from microvascular angina should be treated with beta-blockers as first-line treatment especially if they have elevated heart rate at rest or low-workload exercise and exercise-related symptoms [3].

Between beta-blockers, nebivolol has vasodilatory effects via nitric oxide production, improving angina and exercise capacity. The results of the NIRVANA trial (Study to Evaluate Effect of Nebivolol on Angina in Women With Microvascular Disease—ClinicalTrials.gov Identifier: NCT01665508) will clarify if Nebivolol has any effect on angina, exercise capacity, reducing resource utilization, and improving other measures of artery function in a population of women with microvascular dysfunction.

If beta-blockers fail to improve symptoms, a calcium channel blocker can be added [17].

In patients with proven or suspected coronary spasms, beta-blockers should be avoided, especially nonselective ones. In fact, in a small randomized double-blind trial, propranolol was associated with prolonged angina pectoris attack due to coronary artery spasms [32].

#### Nicorandil and Ranolazine

Other treatment options include nicorandil and ranolazine. Nicorandil is more effective than long-acting nitrates, since it acts either on the vascular smooth muscle cells, and nitric oxide (NO) production, ensuring a greater effect on the microvasculature [11, 33]. On the other side, ranolazine, an antianginal drug that inhibits the late sodium channel and reduces intracellular calcium in cardiomyocytes, can improve ventricular relaxation and facilitate microvascular function [17]. However, studies on the effect of Ranolazine on microvascular dysfunction gave controversial results. In a small study of patients with angina, ranolazine was able to improve CFR compared to placebo (2.54 ± 0.44 vs. 1.91 ± 0.31; *P* = 0.005) [34], but it did not show the same result in another similar size study [35]. In the end, in a large randomized-crossover trial, Ranolazine failed to improve symptoms or find any differences in cardiac magnetic resonance imaging-myocardial perfusion reserve [17, 36].

#### Quinapril

Quinapril, a drug from ACE-inhibitors class, improved microvascular function in women with signs and symptoms of ischemia without obstructive CAD.

Microvascular dysfunction is highly prevalent in woman with chest pain in absence of coronary artery disease: in fact, results from the WISE study showed that 74 (47%) women had subnormal coronary flow velocity reserve suggestive of microvascular dysfunction (mean 2.02 ± 0.38), while 85 (53%) had normal reserve (mean 3.13 ± 0.64) [37]. In women with reduced CFR, a value less than 2.32 is considered the best discriminating threshold for adverse outcomes (event rate > 26.7%; and 2.32 event rate 12.2%; *p* = 0.01) [38].

In a sub-analysis of the WISE study, 78 women with microvascular dysfunction (CFR < 3 after adenosine infusion) and no obstructive CAD > 50% at coronary angiography were randomized to Quinapril or placebo. After 16 weeks, CFR improved more with ACE-I than with placebo (*p* < 0.019). Furthermore, angina frequency improved more with quinapril treatment than with placebo (*p* = 0.037) [17, 39].

#### Nitric Oxide and Cyclic Guanosine Monophosphate Pathway

The nitric oxide pathway is involved in vasodilation since it stimulates guanylate cyclase in vascular smooth muscle to produce cyclic guanosine monophosphate (cGMP). Its vasodilator effect is, then, mediated by a family of cGMP phosphodiesterase [40].

Sildenafil, a phosphodiesterase (PDE) type 5 inhibitor, has proved to increase CFR in an ancillary analysis of WISE study. In 23 symptomatic women with a baseline CFR < 3 and without CAD, 100 mg of oral sildenafil was administered, resulting in a rapid increase of CFR [41]. These encouraging results will need further confirmation with randomized trials [17].

Direct stimulators of soluble guanylate cyclase, such as vericiguat and riociguat, can activate the cGMP pathway independently from NO [42].

Vericiguat has been proved to stimulate soluble guanylate cyclase (sGC) and increase sGC sensitivity to endogenous NO and thus enhance the cGMP pathway. In the SOCRATES-PRESERVED Trial, vericiguat did not change NT-proBNP at 12 weeks compared with placebo, but it was associated with improvements in quality of life in patients with heart failure with preserved ejection fraction (HFpEF) [43].

Riociguat, another sGC stimulator, induces vasodilation and has antifibrotic, anti-proliferative, and anti-inflammatory effects [44]. In the DILATE-1 study, patients with HFpEF and pulmonary hypertension, Riociguat had no significant effect on mean pulmonary artery pressure but improved hemodynamic and echocardiographic parameters [45].

#### Ivabradine

Ivabradine is a heart-rate-lowering agent that acts by selectively and specifically inhibiting the cardiac pacemaker current (If). Ivabradine is recommended by the latest guidelines as an antianginal drug for stable CAD [46], but it also has beneficial effects on microvascular disease where it is demonstrated to improve angina despite its effect on heart rate. However, it is still not known if it has a direct effect on the coronary microvascular function [35, 47, 48]

#### Rho Kinase Inhibitors

Rho-kinases are small proteins that bind guanosine triphosphate and enhance myosin light chain phosphorylation, leading to a modulation of calcium sensitivity of myosin light chain in smooth muscle cells [49]. They seem to play a pathogenetic role in many cardiovascular pathological conditions, such as endothelial dysfunction, hypercontraction of vascular smooth muscle, vasospasm and inflammatory cell accumulation in blood vessel adventitia [50]. Furthermore, the rho-kinase pathway may be implicated in the pathogenetic mechanism that leads to chest pain in patients without obstructive CAD [8]. Rho-kinase inhibitors could be effective in coronary microvascular dysfunction (CMD) when this pathway is involved. In a small study involving 18 women with angina and non-obstructive CAD, who had intracoronary acetylcholine-induced myocardial ischemia but without epicardial coronary spasm, pretreatment with intracoronary fasudil, a rho-kinase inhibitor, ameliorated myocardial ischemia caused by microvascular hypercontraction [17, 51].

#### Endothelin Receptor Antagonists

The endothelium-derived peptide endothelin-1 (ET-1) induces vasoconstriction via smooth muscle endothelin receptor ET(A) activation [52]. ET-1 is known to lead to coronary endothelial dysfunction and it is increased in patients with MVA, where it reduces the time to onset exercise angina [53]. Since ET-1 has a tonic inhibitory effect on myocardial perfusion, it may represent a possible target of treatment in microvascular angina, using endothelin receptor antagonists [17]. In a small trial, 37 patients were randomized to darusentan, an endothelin receptor antagonists (ERA), or placebo. The primary endpoint was the change in rest myocardial perfusion homogeneity evaluated by PET, while the secondary endpoint evaluated variations in absolute myocardial flow and CFR. The results showed that darusentan improved myocardial perfusion and increased the homogeneity perfusion [54].

In another double-blind, randomized, control trial, 47 patients with multiple cardiovascular risk factors, non-obstructive CAD, and coronary endothelial dysfunction were randomized to atrasentan or placebo. After 6 months there, was a significant improvement in coronary blood flow in response to acetylcholine in the atrasentan group compared to the placebo one [55].

To conclude, zibotentan is an ET-A that demonstrated to relax the small blood vessels in patients with MVA. The ongoing “Precision Medicine with Zibotentan in Mirrovascular Angina (PRIZE) trial” (ClinicalTrials.gov Identifier: NCT04097314) will randomize 356 patients with MVA and impaired exercise intolerance to zibotentan or placebo. The primary efficacy outcome is the treadmill exercise time, which is a measure of aerobic capacity that reflects disease severity.

#### Adenosine Active Agents

Dipyridamole, an adenosine active agent, produces selective coronary vasodilatation without systemic hemodynamic effect. For this reason, it has been proposed to evaluate coronary vascular reserve [17]. The study of Marchant et al. enrolled 15 patients with atypical chest pain: the infusion of intracoronary dipyridamole produced a 73% increase in coronary blood flow without significant hemodynamic changes [56]. Furthermore, dipyridamole has been demonstrated to be useful in evaluating CMD in patients with non-obstructive CAD [57, 58]

#### Xanthine Derivates

Xanthine derivates improve exercise capacity in patients with angina by the reduction of myocardial oxygen consumption and by the improvement of myocardial blood flow distribution. In particular, they prevent arteriolar dilatation of adenosine by the inhibition of vascular smooth adenosine-A2 receptors: by this mechanism, coronary blood flow is redistributed towards those ischemic regions where adenosine release is increased [59].

Another important effect of xanthines is their ability to antagonize the stimulation of cardiac nerve pain fibers, leading to an analgesic effect on ischemic chest pain. In a small, randomized trial, 13 patients with typical exertional pain and non-obstructive CAD were randomized to receive oral aminophylline or a placebo. After 3 weeks, oral aminophylline showed a favorable effect either on exercise-induced chest pain threshold (mean 632 s vs 522 s, *P* = 0.004) and on reduction in the total number of episodes of chest pain during the three weeks [60].

In another study, treadmill exercise tests were performed in 14 patients with chest pain and non-obstructive CAD before and after i.v. aminophylline infusion. Aminophylline lengthened the time before the occurrence of ischemia by increasing the ischemia threshold and it also shows a beneficial effect on chest pain induced by exercise [17, 61].

#### Trimetazidine

Trimetazidine is an antianginal agent which is able to modulate cardiac metabolism without altering the hemodynamic functions. Furthermore, it has a positive effect on the inflammatory profile and endothelial function [62]. In a randomized trial of patients with MVA, trimetazidine on top of medical therapy for 3 months improved symptoms, quality of life and exercise tolerance by the improvement of myocardial perfusion and endothelial function [17, 63].

#### *Autologous CD34* + *Stem Cell Therapy*

Stem cell therapy using autologous CD34 + may be a promising therapy for patients with microvascular dysfunction. In fact, CD34 + cells stimulate capillary growth and regeneration of damaged microcirculation in pre-clinical models.

In a small pilot clinical study, 20 patients with non-obstructive CAD, persistent angina and CFR ≤ 2.5 received a single intracoronary infusion of isolated CD34 + cells in the left anterior descending artery. After 6 months, CFR improved (from 2.08 ± 0.32 at baseline to 2.68 ± 0.79, *P* < 0.005), angina frequency decreased (*P* < 0.004), Canadian Cardiovascular Society class improved (*P* < 0.001), and quality of life improved as assessed by the Seattle Angina Questionnaire (*P* ≤ 0.03) and SF-36 (*P* ≤ 0.04) [64].

#### Tricyclic Antidepressant Drugs

Tricyclic antidepressant drugs are a class of medications used for the management and treatment of major depressive disorders. Previous studies have suggested that they may have a beneficial effect on a wide range of conditions associated with chronic pain [65]. In a randomized, double-blind, cross-over trial performed on 18 women with chest pain and non-obstructive CAD, imipramine reduced the incidence of chest pain compared with placebo but failed to improve quality of life [66].

## Conclusion

Coronary (micro)vascular spasm and microvascular dysfunction are clinical entities characterized by high prevalence and clinical representation, burdened by an important clinical and symptomatologic impact. Numerous therapeutic strategies, both innovative and established, are available to optimize treatment and improve the quality of life of these patients (Fig. 1).

## Data Availability

Not applicable.
